# Impact of antenatal antiretroviral drug exposure on the growth of children who are HIV-exposed uninfected: the national South African Prevention of Mother to Child Evaluation cohort study

**DOI:** 10.1186/s12879-022-07847-9

**Published:** 2022-12-06

**Authors:** Vundli Ramokolo, Louise Kuhn, Carl Lombard, Debra Jackson, Ameena E. Goga

**Affiliations:** 1 HIV and Other Infectious Diseases Research Unit, South African Medical Research Council, 1 Francie Van Zyl Drive, Tygerberg, South Africa; 2 Gertrude H. Sergievsky Center, Vagelos College of Physicians and Surgeons, Columbia University Irving Medical Center, New York, NY USA; 3 Department of Epidemiology, Mailman School of Public Health, Columbia University Irving Medical Center, New York, NY USA; 4 Biostatistics Unit, South African Medical Research Council, Tygerberg, South Africa; 5 Division of Epidemiology and Biostatistics Unit, Department of Global Health, University of Stellenbosch, Tygerberg, South Africa; 6 Centre for Maternal Adolescent Reproductive & Child Health (MARCH), London School of Hygiene and Tropical Medicine, London, UK; 7 School of Public Health, University of the Western Cape, Cape Town, South Africa; 8 Department of Paediatrics and Child Health, University of Pretoria, Pretoria, South Africa

**Keywords:** HIV-exposed uninfected, Antiretroviral, Postnatal growth, Children

## Abstract

**Background:**

The relationship between in-utero antiretroviral (ARV) drug exposure and child growth needs further study as current data provide mixed messages. We compared postnatal growth in the first 18-months of life between children who are HIV-exposed uninfected (CHEU) with fetal exposure to ARV drugs (prophylaxis or triple-drug therapy (ART)) and CHEU not exposed to ARVs. We also examined other independent predictors of postnatal growth.

**Methods:**

We analysed data from a national prospective cohort study of 2526 CHEU enrolled at 6-weeks and followed up 3-monthly till 18-months postpartum, between October 2012 and September 2014. Infant anthropometry was measured, and weight-for-age (WAZ) and length-for-age (LAZ) Z-scores calculated. Generalized estimation equation models were used to compare Z-scores between groups.

**Results:**

Among 2526 CHEU, 617 (24.4%) were exposed to ART since -pregnancy (pre-conception ART), 782 (31.0%) to ART commencing post-conception, 879 (34.8%) to maternal ARV prophylaxis (Azidothymidine (AZT)), and 248 (9.8%) had no ARV exposure. In unadjusted analyses, preterm birth rates were higher among CHEU with no ARV exposure than in other groups. Adjusting for infant age, the mean WAZ profile was lower among CHEU exposed to pre-conception ART [-0.13 (95% confidence interval − 0.26; − 0.01)] than the referent AZT prophylaxis group; no differences in mean WAZ profiles were observed for the post-conception ART (− 0.05 (− 0.16; 0.07)), None (− 0.05 (− 0.26; 0.16)) and newly-infected (− 0.18 (− 0.48; 0.13)) groups. Mean LAZ profiles were similar across all groups. In multivariable analyses, mean WAZ and LAZ profiles  for the ARV exposure groups were completely aligned. Several non-ARV factors including child, maternal, and socio-demographic factors independently predicted mean WAZ. These include child male (0.45 (0.35; 0.56)) versus female, higher maternal education grade 7–12 (0.28 (0.14; 0.42) and 12 + (0.36 (0.06; 0.66)) versus ≤ grade7, employment (0.16 (0.04; 0.28) versus unemployment, and household food security (0.17 (0.03; 0.31). Similar predictors were observed for mean LAZ.

**Conclusion:**

Findings provide evidence for initiating all pregnant women living with HIV on ART as fetal exposure had no demonstrable adverse effects on postnatal growth. Several non-HIV-related maternal, child and socio-demographic factors were independently associated with growth, highlighting the need for multi-sectoral interventions. Longer-term monitoring of CHEU children is recommended.

**Supplementary Information:**

The online version contains supplementary material available at 10.1186/s12879-022-07847-9.

## Background

Maternal triple antiretroviral therapy (ART) scale-up for prevention of vertical HIV transmission has improved maternal health and reduced new pediatric infections to < 1% in high income countries [1] and to < 5% in most low and middle income countries with breastfeeding populations [2, 3]. Therefore, most children affected by the HIV pandemic are now HIV-exposed uninfected (CHEU). Furthermore, strengthening ART programs will increase the likelihood that women living with HIV (WLHIV) will conceive subsequent pregnancies on ART. Taken together, the population of children who are HIV- and ART-exposed during critical intrauterine, intrapartum, and postpartum periods will continue to increase as more children are ART-exposed from conception.

There is ongoing discussion about maternal ART effects on birth and postnatal outcomes, with some studies reporting increased adverse birth outcomes, including preterm birth, low birthweight and small for gestational age [4–8], and poorer postnatal growth with *in-utero* ART exposure [9–11]. Given that the current global goal is to move beyond survival of CHEU and ensure that they have optimal health and wellbeing, more data are required on short and long-term health outcomes of these children. Data from South Africa are particularly important as it is home to a quarter of CHEU globally (estimated at 3.8 million in 2019) [12]. The country also has the largest ART programme [13], and antenatal HIV prevalence has remained around 30% for the past decade, though incident pediatric HIV infections have declined [14]. Furthermore, relationships between HIV and ART exposure and postnatal growth need further exploration in a context of endemic undernutrition and overnutrition [15, 16], childhood illnesses such as diarrhoea [17], syndemic interactions [18], including non-HIV related risk factors such as poverty and inequality [19].

We compared postnatal growth in the first 18-months of life between CHEU with fetal exposure to antiretroviral (ARV) drugs as prophylaxis or as triple-drug combinations intended as therapy (ART) and CHEU without fetal exposure to ARVs. We also examined independent associations of other maternal, child, household, and socio-demographic factors with postnatal growth.

## Methods

### Study design, setting and methods

The 2012 South African Prevention of Mother to Child Transmission of HIV Evaluation (SA-PMTCT-E) was a nationally-representative health facility-based cross-sectional study that enrolled 6-week (range 4–8 weeks) old infants attending immunization clinics in 9 South African provinces, with the primary aim to measure national PMTCT programme’s early effectiveness [20, 21]. In brief, nurse data collectors sampled mother-infant pairs systematically in large facilities (where participants were recruited at selected fixed intervals based on the target sample) or consecutively in small facilities (where all eligible participants were recruited until target sample obtained) during 6-week immunization visits. Sick infants needing emergency care or hospitalization were excluded.

Infant HIV exposure was used as a marker of maternal HIV status. This was established through antibody testing [Genscreen HIV1/2 Ab EIA (enzyme immunoassay) Bio-Rad and confirmatory Vironostika HIV Uni-form II plus O, bioMérieux, France] on infant dried blood spots. Infant HIV infection status was assessed using polymerase chain reaction (PCR) testing [COBAS AmpliPrep/COBAS TaqMan assay, Roche, New Jersey] on the same dried blood spots [2].

Infants whose mothers reported living with HIV or infants with a positive 6-week HIV antibody test regardless of maternal self-reported HIV status, were eligible for recruitment into a prospective cohort study, nested within a national cross-sectional survey, to measure vertical transmission risk until 18-months postpartum. Recruitment was from 29 October 2012 to 31 May 2013, with follow-up until September 2014. Detailed cohort methods are described elsewhere [2]. In brief, consenting mother-infant pairs were followed at 3, 6, 9, 12, 15 and 18-months during scheduled facility visits coinciding with routine childcare appointments. Infant blood specimens collected between 6-weeks and 15-months underwent afore-mentioned diagnostic tests. At the 18-month visit, study nurses documented results from routinely-administered HIV-1 rapid test (SD Bioline HIV 1/2 3.0 Titma Health, Pty) on the child.

### Exposure measures and covariates

Our primary exposure of interest was fetal exposure to maternal ARV, based on self-reported maternal ARV drug use data obtained using a structured questionnaire. During the study period, the national PMTCT programme was implementing CD4 count criteria-based life-long maternal ART initiation (CD4 cell counts ≤ 350 cells/mm^3^) and infant prophylaxis (World Health Organization (WHO) PMTCT policy Option A: 1 April 2010- 31 March 2013). Women with CD4 cell counts > 350 cells/mm^3^ were given Azidothymidine (AZT) prophylaxis from 14 weeks gestation. In April 2013, the PMTCT programme transitioned to lifelong ART for all pregnant and lactating WLHIV (WHO PMTCT Option B+) [22, 23]. ART regimens generally consisted of Nevirapine, Tenofovir, and Lamivudine or Emtricitabine [24]. We classified reported maternal ARV exposure as follows in analyses: (1) “pre-conception ART” when ART was started before pregnancy, (2) “post-conception ART” when ART was started during pregnancy, (3) “AZT only” when only AZT-based prophylaxis was given, (4) “None” when a known WLHIV took no ARV drugs during pregnancy, and (5) “Newly-infected” when women reported to have been HIV-negative during pregnancy but their children’s 6-week HIV antibody test result was positive.

Study questionnaires also included questions on self-reported 24-h and 1-week child feeding practices, maternal (Tuberculosis (TB), HIV, CD4 count, syphilis) and child (coughing, diarrhea) morbidity and treatment, maternal obstetric history, socio-demographics characteristics, and peripartum community social support at each time point.

### Outcomes

Trained nurse data collectors collected anthropometric data using standardised procedures based on WHO guidelines [25]. Child weight was measured using calibrated A&D personal precision weight scales (UC-321) and length using SECA portable baby length boards (SCA417BLM). Measurements were recorded in kilograms or centimeters to two decimal places. Birthweight, birth length, and gestational age were extracted from participant-held health booklets.

We defined low birthweight (LBW) as birthweight < 2.5 kg; preterm birth (PTB) as birth before 37 completed weeks gestation; and small for gestational age (SGA) as birthweight for gestational age Z-score below − 1.28 [26]. We estimated birthweight and length–for-gestational-age Z-scores using Intergrowth international standards for assessing newborn size for term and pre-term infants [27] and LMSgrowth [28], and excluded gestational ages outside of range for these standards (20 to 24 weeks). We estimated weight-for-age (WAZ), weight-for-length (WLZ), and length-for-age (LAZ) Z-scores at each postnatal timepoint using WHO growth standards [29]. We considered infants as underweight and stunted if their WAZ and LAZ were below -2 standard deviations respectively [25].

Anthropometric measurements and Z-scores were flagged based on criteria (Additional file 1: Box 1), and set to missing if no plausible explanation was established.

### Statistical analysis

We performed statistical analyses using STATA standard edition version 15. We calculated frequencies for categorical variables and means (standard deviations) or medians (inter quartile range) for continuous variables. Proportions were compared using Pearson chi-squared test while F-test was used for comparing means. Generalized estimation equations, with a gaussian distribution, were used for univariable and multivariable regression analyses to account for correlations between repeated anthropometric measurements within the same participant. Model covariates were selected based on literature (Additional file 1: Fig. S1) [30] and quasi-likelihood under an independence model criterion. These criteria were also used to inform the best fitting final multivariable models, and to select the independent correlation structure [31]. Mean WAZ and LAZ over the study period were modelled on exposure group, infant age (in months) and important covariates including maternal age, education, employment status, syphilis and TB status, mode and place of delivery, birth attendant, household food security, housing type (brick or non-brick), access to flush toilet and electricity, infant breastfeeding, sex and geographic location. These covariates were treated as potential confounders in the adjusted models assessing the association between foetal ARV exposure and postnatal growth. They were also included as predictors in the multivariable predictive models assessing factors independently associated with study outcomes. Presence of effect measure modification was also explored. We excluded variables that were affected by exposures of interest and shared common causes with outcomes (i.e., LBW, SGA and PTB) from models to minimize bias introduced by adjustment of potential mediators in the presence of unmeasured common causes [32, 33]. We did not include survey sampling weights in final analyses because this adjustment (1) did not change the findings and (2) generally increases standard errors. Instead, we added province in models to adjust for the survey structure. Point estimates were calculated with 95% confidence intervals. Analyses only included CHEU and children were censored if they tested HIV PCR positive at last HIV negative PCR result or died (censored at time point when death was reported). Although statistical testing was performed at the 5% statistical significance level, results were interpreted primarily based on the precision of the estimates.

## Results

### Participant characteristics

A total of 9120 children were recruited at the 6-week visit, of which 2877 were classified as CHEU children. 2526 CHEU were retained for analysis after excluding 67 CHEU with positive PCR results at 6-weeks or earlier, 25 CHEU with rejected 6-week PCR results, 13 CHEU with equivocal 6-week PCR results, 77 CHEU with missing 6-week PCR results, 70 CHEU with no consent for follow-up, 81 CHEU with missing ARV exposure data, and 18 CHEU with no in-utero HIV and ARV exposure data (Additional file 1: Fig. S2). Thirty CHEU who tested PCR positive postnatally were censored. Among the CHEU, 617 (24.4%) were born to WLHIV who initiated ART pre-conception, 782 (31.0%) were born to WLHIV who initiated ART post-conception, 879 (34.8%) were exposed only to AZT as prophylaxis, 189 (7.5%) were born to WLHIV who did not receive any ARV drugs, and 59 (2.3%) were born to women who reported being HIV-negative and had no ARV drugs during pregnancy, but whose children tested antibody positive at 6 weeks. ARV exposure status was treated as a constant as few women moved across exposure groups during follow-up (Additional file 1: Table S3), making possibility of misclassification bias minimal.

Baseline socio-demographic characteristics of exposure groups are shown in Table 1. WLHIV who initiated ART pre-conception were generally older, multiparous, and employed. They had more ANC visits, caesarian sections (c-section), and hospital-based deliveries, and reported higher rates of TB or syphilis than other exposure groups. WLHIV who initiated ART post-conception had the lowest median (interquartile rage) CD4 cell count [271 (186–340)] followed by those who initiated ART post-conception [350 (250–500)]. Mothers of children exposed to AZT had higher education levels, more births attended by a nurse and a higher median CD4 count than women in ART groups. Newly-infected mothers were younger, primiparous, most likely to have household access to electricity and least likely to report TB or syphilis during pregnancy. Peripartum social support, infant Nevirapine prophylaxis, household piped water or food insecurity, and the child’s sex did not differ between groups.

**Table 1:** Baseline maternal and child characteristics at 6-weeks postpartum by peripartum antiretroviral exposure, 2012–2014, South Africa

Maternal characteristic	Total N = 2526	Pre-conception ART N = 617	Post-conception ART N = 782	AZT prophylaxis N = 879	None N = 189	Newly infected mothers N = 59
	n (%)	n (%)	n (%)	n (%)	n (%)	n (%)
Age (years)						
13–19	109 (4.32)	13 (2.11)	23 (2.94)	58 (6.60)	11 (5.82)	4 (6.78)
20–35	2063 (81.67)	449 (72.77)	668 (85.42)	738 (83.96)	160 (84.66)	48 (81.36)
35+	354 (14.01)	155 (25.12)	91 (11.64)	83 (9.44)	18 (9.52)	7 (11.86)
Parity						
Primipara	588 (23.28)	95 (15.40)	188 (24.04)	255 (29.01)	31 (16.40)	19 (32.20)
Multipara	1938 (76.72)	522 (84.60)	594 (75.96)	594 (70.99)	158 (83.60)	40 (67.80)
Education (grade)						
None-grade 7	490 (19.40)	142 (23.01)	138 (17.65)	146 (16.61)	51 (26.98)	13 (22.03)
7–12	1935 (76.60)	456 (73.91)	617 (78.90)	687 (78.16)	131 (69.31)	44 (74.58)
12+	101 (4.25)	19 (3.08)	27 (3.45)	46 (5.23)	7 (3.70)	2 (4.25)
Delivery mode						
Vaginal	1920 (76.16)	456 (73.91)	577 (74.16)	686 (78.04)	154 (81.91)	47 (76.16)
C-section	601 (23.84)	161 (26.09)	201 (25.84)	193 (21.96)	34 (18.09)	12 (23.84)
Missing	5	0	4	0	1	0
CD4 count at 6 weeks, n, median cells/mm^3^(IQR)	1587, 373 (268–508)	417, 350 (250–500)	514, 271 (186–340)	562, 480 (390–598)	93, 495(367–600)	1, 734 (734–734)
ANC visits						
1–5	1031 (60.15)	247 (56.39)	303 (58.83)	359 (60.95)	90 (69.23)	32 (76.19)
5+	683 (39.85)	191 (43.61)	212 (41.17)	230 (39.05)	40 (30.77)	10 (23.81)
Missing	812	179	267	290	59	17
Syphilis (antenatal)						
Positive	159 (8.91)	52 (11.82)	57 (10.12)	42 (6.65)	8 (7.21)	0 (0)
Negative	1625 (91.09)	388 (88.18)	506 (89.88)	590 (93.35)	103 (92.79)	38 (100)
Missing	742	177	219	247	78	21
Current tuberculosis						
Yes	128 (5.09)	59 (9.61)	42 (5.40)	21 (2.39)	5 (2.66)	1 (1.69)
No	2388 (94.91)	555 (90.39)	736 (94.60)	856 (97.61)	183 (97.34)	58 (98.31)
Missing	10	3	4	2	1	0
Place of delivery						
Hospital	1973 (78.11)	497 (80.55)	618 (79.03)	683 (77.70)	128 (67.72)	47 (79.66)
Clinic	449 (17.78)	95 (15.40)	148 (18.93)	159 (18.09)	39 (20.63)	8 (13.56)
Home	104 (4.12)	25 (4.05)	16 (2.05)	37 (4.21)	22 (11.64)	4 (6.78)
Birth attendant						
Doctor	728 (28.82)	186 (30.15)	238 (30.43)	243 (27.65)	43 (22.75)	18 (30.51)
Nurse	1704 (67.46)	409 (66.29)	531 (67.90)	602 (68.49)	125 (66.14)	37 (62.71)
TBA	94 (3.72)	22 (3.57)	13 (1.66)	34 (3.87)	21 (11.11)	4 (6.78)
Peri-partum social support						
Yes	1969 (77.95)	488 (79.09)	604 (77.24)	676 (76.91)	153 (80.95)	48 (81.36)
No	557 (22.05)	129 (20.91)	178 (22.76)	203 (23.09)	36 (19.05)	11 (18.64)
Mother employed						
Yes	501 (19.83)	143 (23.18)	168 (21.48)	153 (17.41)	25 (13.23)	12 (20.34)
No	2025 (80.17)	474 (76.82)	614 (78.52)	726 (82.59)	164 (86.77)	47 (79.66)
Male	1285 (50.87)	310 (50.24)	386 (49.36)	465 (52.90)	91 (48.15)	33 (55.93)
Female	1241 (49.13)	307 (49.76)	396 (50.64)	414 (47.10)	98 (51.85)	26 (44.07)
Piped water						
Yes	1913 (75.73)	479 (77.63)	596 (76.21)	660 (75.09)	139 (73.54)	39 (66.10)
No	613 (24.27)	138 (22.37)	186 (23.79)	219 (24.91)	50 (26.46)	20 (33.90)
Flush toilet						
Yes	1350 (53.44)	359 (58.18)	426 (54.48)	466 (53.01)	69 (36.51)	30 (50.85)
No	1176 (46.56)	258 (41.82)	356 (45.52)	413 (46.99)	120 (63.49)	29 (49.15)
Electricity						
Yes	2377 (94.10)	585 (94.81)	747 (95.52)	815 (92.72)	173 (91.53)	57 (96.61)
No	149 (5.90)	32 (5.19)	35 (4.48)	64 (7.28)	16 (8.47)	2 (3.39)
No food in house						
Yes	496 (19.64)	139 (22.53)	143 (18.29)	174 (19.80)	31 (16.40)	9 (15.25)
No	2027 (80.25)	478 (77.47)	638 (81.59)	703 (79.98)	158 (83.60)	50 (84.75)
Child sex						
Male	1285 (50.87)	310 (50.24)	386 (49.36)	465 (52.90)	91 (48.15)	33 (55.93)
Female	1241 (49.13)	307 (49.76)	396 (50.64)	414 (47.10)	98 (51.85)	26 (44.07)
Breastfed at 6 weeks						
Yes	1647 (66.84)	374 (61.61)	504 (65.88)	589 (68.97)	131 (71.98)	49 (87.50)

ANC: antenatal care; ART: antiretroviral therapy (Maternal ART regimens generally consisted of Tenofovir, Lamivudine or Emtricitabine and Nevirapine); AZT: Azidothymidine; HEU: HIV exposed uninfected; None: children with no foetal ARV exposure; SD: standard deviation

### Adverse birth outcomes

Mean birthweight, birthweight-for-gestational age Z-score and birth length did not differ between ARV exposure groups (Table 2). However, the proportion of PTB was higher among children born to newly-infected women than in other groups.

**Table 2:** Preterm, low birth weight and small for gestational age by peripartum antiretroviral exposure, 2012–2014, South Africa

Birth outcome	Total N = 2526	Pre-conception ART N = 617	Post-conception ART N = 782	AZT prophylaxis N = 879	None N = 189	Newly-infected mothers N = 59	P-value*
	n (%)	n (%)	n (%)	n (%)	n (%)	n (%)	
Preterm							
Yes	259 (14.4)	66 (14.7)	65 (11.5)	92 (14.8)	24 (20.0)	12 (26.1)	0.02
No	1543 (85.6)	383 (85.3)	499 (88.5)	531 (85.2)	96 (80.0)	34 (73.9)	
Missing	724	168	218	256	69	13	
Low birthweight							
Yes	352 (14.3)	87 (14.4)	119 (15.6)	102 (11.9)	35 (19.1)	9 (16.1)	0.07
No	2114 (85.7)	517 (85.6)	646 (84.4)	756 (88.1)	148 (80.9)	47 (83.9)	
Missing	60	13	17	21	6	3	
Birthweight (kg) n [mean ± SD]	2465, 3.00 (0.54)	604, 2.96 (0.51)	765, 2.99 (0.54)	857, 3.04 (0.52)	183, 2.99 (0.63)	56, 2.99 (0.67)	0.08**
Small for gestational age							
Yes	311 (12.6)	85 (14.1)	108 (14.1)	95 (11.1)	15 (8.2)	8 (14.3)	0.09
No	2154 (87.4)	519 (85.9)	657 (85.9)	762 (88.9)	168 (91.8)	48 (85.7)	
Missing	61	13	17	22	6	3	
Birthweight for gestational age Z-score n [mean ± SD]	1777, − 0.16 (1.22)	443, − 0.21 (1.27)	559, − 0.23 (1.20)	610, − 0.10 (1.20)	119, − 0.03 (1.22)	46, 0.03 (1.33)	0.19**
Birth length (cm) n [mean ± SD]	2401, 49.30 (3.52)	590, 49.20 (3.29)	746, 49.24 (3.66)	839, 49.46 (3.56)	174, 49.19 (3.44)	52, 48.85 (3.60)	0.47**

^*^P-value from chi-squared test **Overall p-value from F-test; SD, kg, AZT

ANC: antenatal care; ART: antiretroviral therapy (Maternal ART regimens generally consisted of Tenofovir, Lamivudine or Emtricitabine and Nevirapine); AZT: Azidothymidine; HEU: HIV exposed uninfected; None, children with no foetal ARV exposure; SD: standard deviation

### Mean WAZ (6 weeks to 18 months)

**Fig. 1:**
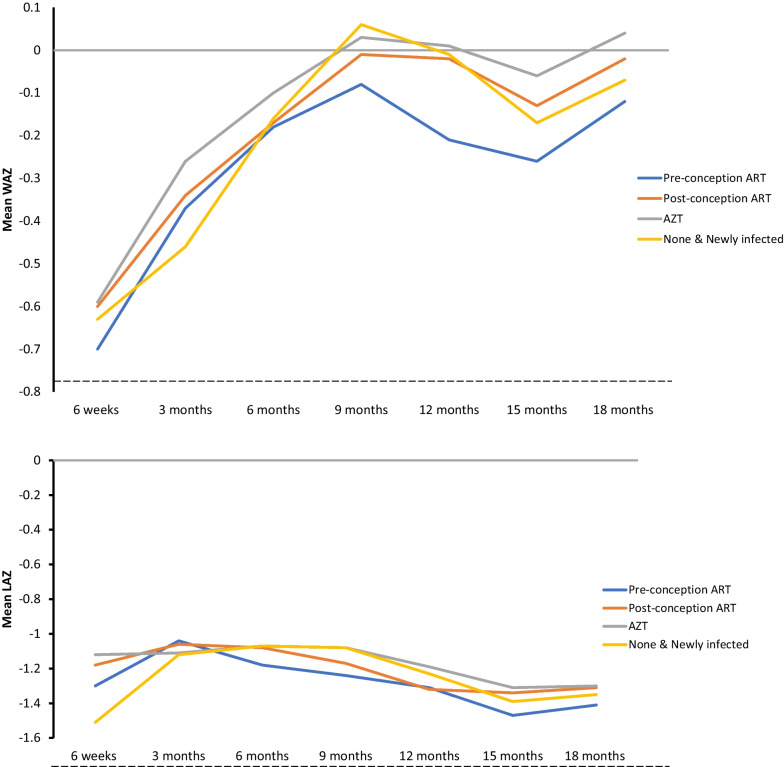
Mean weight-for-age and length-for-age Z-scores from 6-weeks to 18-months by antiretroviral exposure groups, 2012–2014, South Africa. ANC: antenatal care; ART: antiretroviral therapy (Maternal ART regimens generally consisted of Tenofovir, Lamivudine or Emtricitabine and Nevirapine); AZT: Azidothymidine; HEU: HIV exposed uninfected; None: children with no foetal ARV exposure

In unadjusted analyses, CHEU exposed to ART pre-conception tended to have the lowest mean WAZ profile while CHEU exposed to *in-utero* AZT tended to have the highest mean WAZ profile (Fig. 1). In an analysis only adjusting for child age, the mean (95% confidence interval) WAZ profile for children born to WLHIV who initiated ART pre-conception [− 0.13 (− 0.26; − 0.01)] was lower than for CHEU exposed to AZT (Table 3). Although this association between ART and mean WAZ remained after adjusting for several covariates, it was attenuated in the final multivariable model including provincial location (Table 3). Mean WAZ profiles for CHEU with foetal exposure to ART post-conception and those exposed to AZT were parallel. These profiles were also similar between the ARV exposed and unexposed groups (Table 3 and Fig. 1). Several socio-economic, child and maternal factors were independently associated with an increase in mean WAZ in the predictive multivariable model, including having a male child, older maternal age, higher maternal education, maternal employment, c-section delivery, birth attendance by a nurse, household electricity, and household food security. Maternal TB infection, home-based delivery, and breastfeeding were associated with lower mean WAZ. Mean WAZ also differed by geographical location with provinces such as Free State, Gauteng, Northern Cape, North West and Western Cape showing a lower mean WAZ than Eastern Cape (Table 4). Analyses stratified by province showed mean differences in WAZ between ARV groups in some provinces (Free State, Mpumalanga, and North West) but not in others (Gauteng, Eastern Cape, KwaZulu-Natal, Limpopo, Northern Cape and Western Cape), yielded inconsistent associations between ARV exposure and mean WAZ (data not shown). The proportion of underweight children did not differ between exposure groups (Additional file 1: Table S1).

**Table 3:** Mean weight-for-age and length-for-age Z-scores and unadjusted Generalized Estimating Equations regression model for HIV exposed uninfected children, 2012–2014, South Africa

ARV groups	6-weeks	3 months	6 months	9 months	12 months	15 months	18-months	Unadjusted GEE Model**	Adjusted GEE model**
WAZ	n/N, mean (SD)	n/N, mean (SD)	n/N, mean (SD)	n/N, mean (SD)	n/N, mean (SD)	n/N, mean (SD)	n/N, mean (SD)	β(95 CI%)	
Pre-conception ART (N = 617)	583/617, − 0.70 (1.34)	436/450, − 0.37 (1.21)	449/454, − 0.18 (1.32)	434/442, − 0.08 (1.35)	438/441, − 0.21 (1.36)	440/443, − 0.26 (1.34)	464/465, − 0.12 (1.27)	− 0.13 (− 0.26; − 0.01)	− 0.08 (− 0.20; 0.04)
Post-conception ART (N = 782)	735/782, − 0.60 (1.28)	532/555, − 0.34 (1.28)	506/512, − 0.17 (1.29)	504/509, − 0.01 (1.32)	492/498, − 0.02 (1.31)	488/492, − 0.13 (1.2)	535/539, − 0.02 (1.23)	− 0.05 (− 0.16; 0.07)	− 0.05 (− 0.16; 0.06)
AZT (N = 879)	836/879, − 0.59 (1.31)	576/612, − 0.26 (1.26)	548/554, − 0.11 (1.23)	523/528, 0.03 (1.21)	507/522, 0.01 (1.27)	530/535, − 0.06 (1.23)	568/574, 0.03 (1.18)	Ref	Ref
None (N = 189)	165/189, − 0.57 (1.55)	108/113, − 0.47 (1.51)	101/102, − 0.17 (1.27)	100/100, 0.06 (1.44)	95/95, 0.02 (1.37)	106/107, − 0.14 (1.26)	106/107, − 0.19 (1.17)	− 005 (− 0.26; 0.16)	0.03 (− 0.17; 0.22)
Newly infected mothers (N = 59)	51/59, − 0.82 (1.59)	5/6, − 0.11 (2.24)	16/16, − 0.10 (1.24)	27/29, 0.04 (1.11)	33/33, − 0.08 (1.08)	34/35, − 0.26 (1.22)	38/38, − 0.21 (1.15)	− 0.18 (− 0.48; 0.13)	− 0.06 (− 0.35; 0.23)

ARV groups	6-weeks	3 months	6 months	9 months	12 months	15 months	18-months	Unadjusted GEE Model***	Adjusted GEE model***
LAZ	n/N, mean (SD)	n/N, mean (SD)	n/N, mean (SD)	n/N, mean (SD)	n/N, mean (SD)	n/N, mean (SD)	n/N, mean (SD)	Β (95 CI%)	Β (95 CI%)
Pre-conception ART (N = 617)	224/617, − 1.27 (1.95)	346/450,− 1.04 (1.77)	431/454,− 1.18 (1.60)	427/442,− 1.24 (1.73)	426/441,− 1.31 (1.51)	431/443,− 1.47 (1.52)	463/465,− 1.41 (1.57)	− 0.11 (− 0.25;0.04)	− 0.09 (− 0.23; 0.04)
Post-conception ART (N = 782)	326/782, − 1.18 (1.65)	439/555, − 1.06 (2.08)	484/512, − 1.08 (1.64)	491/509, − 1.17 (1.57)	481/498, − 1.31 (1.51)	479/492, − 1.34 (1.48)	529/539, − 1.31 (1.64)	− 0.04 (− 017;0.09)	− 0.07 (− 0.19; 0.06)
AZT (N = 879)	349/879,− 1.12 (1.84)	469/612.− 1.11 (1.85)	533/554,− 1.07 (1.64)	505/528,− 1.09 (1.60)	503/522,− 1.19 (1.45)	525/535,− 1.31 (1.45)	567/574,− 1.30 (1.49)	Ref	Ref
None (N = 189)	67/189,− 1.59 (1.73)	84/113, − 1.11 (1.74)	100/102, − 1.21 (1.64)	96/100, − 1.13 (1.71)	92/95, − 1.23 (1.50)	107/107, − 1.44 (1.58)	107/107, − 1.43 (1.53)	− 0.12 (− 0.36;0.11)	− 0.05 (− 0.27; 0.18)
Newly infected mothers (N = 59)	31/59, − 1.33 (1.82)	3/6, − 1.52 (1.29)	15/16, − 0.17 (1.46)	24/29, − 0.90 (1.43)	32/33, − 1.25 (1.56)	35/35, − 1.23 (1.36)	38/38,− 1.12 (1.35)	0.10 (− 0.24;0.44)	0.15 (− 0.20; 0.51)

**The values in the Generalized Estimation Equation model are regression coefficients (95% CI) which describe the mean WAZ. The model included 2496 HIV exposed uninfected children with 12,109 observations and was adjusted for child age (months). Adjusted model included child age and sex, maternal age, education, employment, delivery mode, TB, place of delivery, birth attendant, food security, type of housing, flush toilet, electricity, breastfeeding and province

***The values in the Generalized Estimation Equation model are regression coefficients (95% CI) which describe the mean LAZ. The model included 2257 HIV exposed uninfected children with 10,256 observations and was adjusted for child age (months). Adjusted model included child age and sex, maternal age, education, employment, delivery mode, TB, place of delivery, food security, toilet, breastfeeding and province

ART: antiretroviral therapy (Maternal ART regimens generally consisted of Tenofovir, Lamivudine or Emtricitabine and Nevirapine); AZT: Azidothymidine; None: children with no foetal ARV exposure; Ref: reference; SD: standard deviation; WAZ: weight-for-age Z-score

**Table 4:** Multivariable generalized estimating equations regression for predictors of weight-for-age Z-scores in HIV exposed uninfected children, 2012–2014, South Africa

Variable	N^b^	Model 1^a^		Model 2	
		β	95% CI	β	95% CI
ARV group					
Pre-conception ART	613	− 0.14	− 0.25; − 0.02	− 0.08	− 0.20; 0.04
Post-conception ART	773	− 0.09	− 0.20; 0.03	− 0.05	− 0.16; 0.06
None	180	− 0.01	− 0.20; 0.20	0.03	− 0.17; 0.22
Newly infected	59	− 0.06	− 0.36; 0.23	− 0.06	− 0.35; 0.23
AZT	871	Ref	–	Ref	–
Age (months)	2492	0.03	0.03; 0.03	0.03	0.03; 0.03
Sex					
Male	1272	0.26	0.17; 0.35	0.27	0.18; 0.35
Female	1224	Ref	–	Ref	–
Maternal age (years)					
20–35	2040	0.22	− 0.01; 0.44	0.22	0.00; 0.44
35+	349	0.18	− 0.08; 0.44	0.19	− 0.06; 0.44
14–19	107	Ref	–	Ref	–
Maternal education (grade)					
7–12	1917	0.26	0.13; 0.39	0.24	0.11; 0.37
12+	100	0.29	0.01; 0.57	0.23	− 0.06; 0.51
≤ 7	479	Ref	–	Ref	–
Delivery mode					
C-section	589	0.26	0.05; 0.47	0.24	0.04; 0.45
Vaginal	1902	Ref	–	Ref	–
Maternal TB					
Yes	128	− 0.37	− 0.59; − 0.15	− 0.38	− 0.60; − 0.16
No	2358	Ref	–	Ref	–
Place of delivery					
Clinic	446	0.02	− 0.10; 0.14	0.04	− 0.08; 0.16
Home	100	− 0.93	− 1.65; − 0.18	− 1.00	− 1.78; − 0.21
Hospital	1950	Ref	–	Ref	–
Birth attendant					
Nurse	1690	0.23	0.03; 0.42	0.21	0.02; 0.41
TBA	90	0.63	− 0.13; 1.40	0.68	− 0.15; 1.52
Doctor	716	Ref	–	Ref	–
Food insecurity at 6-weeks					
No	2003	0.13	0.01; 0.25	0.09	− 0.03; 0.21
Yes	490	Ref	–	Ref	–
Brick house					
Yes	1830	0.08	− 0.03; 0.18	0.06	− 0.05; 0.18
No	666	Ref	–	Ref	–
Flush toilet					
Yes	1339	− 0.06	− 0.16; 0.04	0.04	− 0.08; 0.15
No	1157	Ref	–	Ref	–
Electricity					
Yes	2348	0.11	− 0.08; 0.29	0.20	0.01; 0.39
No	148	Ref	–	Ref	–
Breastfeeding at 6-weeks					
Yes	1647	− 0.22	− 0.32; − 0.12	− 0.15	− 0.25; − 0.05
No	817	Ref	–	Ref	–
Mother employed					
Yes	495	0.16	0.04; 0.28	0.16	0.04; 0.28
No	2001	Ref			
Province					
Free State	256			− 0.51	− 0.71; − 0.30
Gauteng	499			− 0.21	− 0.40; − 0.02
KwaZulu-Natal	418			0.01	− 0.18; 0.20
Limpopo	256			− 0.15	− 0.36; 0.06
Mpumalanga	292			− 0.18	− 0.38; 0.01
Northern Cape	78			− 0.92	− 1.26; − 0.58
North West	214			− 0.39	− 0.61; − 0.16
Western Cape	234			− 0.30	− 0.52; − 0.09
Eastern Cape	249			Ref	–

Model 1 included 2496 HIV exposed uninfected children with 12,109 observations. Thirty children were excluded because they did not have WAZ data

Model 2 included 2496 HIV exposed uninfected children with 12,109 observations. Model included province (geographical location)

^a^The values in the models are Generalized Estimation Equation regression coefficients (95% CI) which describe the mean WAZ. ART: antiretroviral therapy (Maternal ART regimens generally consisted of Tenofovir, Lamivudine or Emtricitabine and Nevirapine); AZT: Azidothymidine; HEU; HIV exposed but uninfected; None: children with no foetal ARV exposure; Ref: Reference category; SD: standard deviation; TBA: traditional birth attendant; WAZ: weight-for-age Z-score. Only variables that had a significant association with WAZ in the bivariate analysis were included the final models. The multivariable model with the lowest quasilikelihood under the independence model criteria (QIC) was selected as the final model

^b^Categories for missing data are not shown in the table

### Mean LAZ (6 weeks to 18 months)

 The mean LAZ profiles of the exposure groups overlapped (in time and means) in the unadjusted and adjusted analyses (Fig. 1, Table 3). Several factors were independently associated with a higher mean LAZ in the multivariable model, including having a male child, higher maternal education and maternal employment (Table 5). In contrast home-based delivery, breastfeeding and household food insecurity were associated with lower mean LAZ. Furthermore, CHEU living in provinces except Western Cape had lower mean LAZ than CHEU in Eastern Cape. Although analyses stratified by province showed mean differences in LAZ between ARV groups in some provinces (Eastern Cape, Free State, Gauteng, Limpopo and Mpumalanga), these differences were inconsistent across provinces. There was therefore no clear evidence of an association between ARV exposure and mean LAZ in analyses stratified by province. Evidence showed consistent associations between mean LAZ and socio-demographic and health factors such as maternal education and employment status, place of delivery, food security, breastfeeding, child sex and maternal TB status within provinces (data not shown). The proportion of stunted children also did not differ between exposure groups (Additional file 1: Table S2).

**Table 5:** Multivariable generalized estimating equations regression for predictors of length-for-age Z-scores in HIV exposed uninfected children, 2012–2014, South Africa

Variable	N	Model 1^a^		Model 2	
		β	95% CI	β	95% CI
ARV group					
Pre-conception ART	568	− 0.11	− 0.24; 0.03	− 0.09	− 0.23; 0.04
Post-conception ART	704	− 0.07	− 0.20; 0.06	− 0.07	− 0.19; 0.06
None	157	− 0.06	− 0.30; 0.18	− 0.05	− 0.27; 0.18
Newly infected	50	0.21	− 0.13;0.56	0.15	− 0.20; 0.51
AZT	778	Ref		Ref	
Age (months)	2253	− 0.02	− 0.03; − 0.01	− 0.02	− 0.02; − 0.01
Sex					
Male	1143	0.45	0.35; 0.56	0.44	0.34; 0.54
Female	1114	Ref		Ref	
Maternal education (grade)					
7–12	1734	0.28	0.14; 0.42	0.29	0.16; 0.43
12+	97	0.36	0.06; 0.66	0.30	0.01; 0.60
≤ 7	426	Ref		Ref	
Maternal TB					
Yes	114	− 0.27	− 0.55; 0.01	− 0.15	− 0.34; 0.12
No	2134	Ref		Ref	
Place of delivery					
Clinic	403	− 0.09	− 0.23; 0.04	− 0.01	− 0.13; 0.12
Home	89	− 0.59	− 0.85; − 0.33	− 0.63	− 0.87; − 0.39
Hospital	1765	Ref		Ref	
Food insecurity					
No	1819	0.17	0.03; 0.31	0.13	− 0.01; 0.26
Yes	436	Ref		Ref	
Flush toilet					
Yes	1210	0.06	− 0.05; 0.17	0.04	− 0.08; 0.16
No	1047	Ref			
Mother employed					
Yes	1810	0.10	− 0.03; 0.22	0.04	− 0.08; 0.16
No	447	Ref			
Breastfeeding at 6 weeks					
Yes	727	− 0.23	− 0.34; − 0.11	− 0.17	− 0.27; − 0.06
No	1472	Ref			
Province					
Free State	223			− 0.29	− 0.52; − 0.06
Gauteng	221			− 0.38	− 0.59; − 0.18
KwaZulu-Natal	457			− 0.52	− 0.74; − 0.31
Limpopo	370			− 0.41	− 0.63; − 0.18
Mpumalanga	235			− 0.33	− 0.55; − 0.11
Northern Cape	251			− 0.40	− 0.72; − 0.07
North West	77			− 1.11	− 1.36; − 0.86
Western Cape	193			− 0.20	− 0.43; 0.03
Eastern Cape	230			Ref	

Model 1 included all variables that were significantly associated with LAZ in the bivariate analysis and did not adjust for province. The model included 2257 HIV exposed uninfected children with 10,256 observations. 269 children were excluded because they did not have LAZ data

Model 2 included all variables that were significantly associated with LAZ in the bivariate analysis and adjusted for province (geographical location. The model included 2257 HIV exposed uninfected children with 10,256 observations

^a^The values in the models are Generalized Estimation Equation regression coefficients (95% CI) which describe the mean LAZ. ART: antiretroviral therapy (Maternal ART regimens generally consisted of Tenofovir, Lamivudine or Emtricitabine and Nevirapine); AZT: Azidothymidine; HEU; HIV exposed but uninfected; LAZ: length-for-age Z-score; None: children with no foetal ARV exposure; Ref; Reference category; SD: standard deviation; TBA: traditional birth attendant. Only variables that had a significant association with LAZ in the bivariate analysis were included the final models. The multivariable model with the lowest quasilikelihood under the independence model criteria (QIC) was selected as the final model

## Discussion

Short and long-term health outcomes of CHEU require close monitoring, particularly in settings with high antenatal HIV prevalence and ART coverage such as South Africa. In this national prospective cohort study of CHEU, we found no evidence supporting a detrimental effect of fetal exposure to maternal ARV on birthweight, birthweight-for-gestational age Z-score and birth length. The PTB proportion tended to be higher among children born to women who had received no ART, particularly those who were thought to be newly-infected. Women who had not received ART also reported fewer ANC visits and more frequent home deliveries. The lower antenatal attendance and unmanaged HIV could have contributed to the higher PTB among this group. These findings emphasize the importance of closing HIV testing and ART initiation gaps in healthcare services.

In unadjusted analysis, the mean WAZ profile was lower among children with fetal exposure to maternal ART from pre-conception than in children with foetal exposure to maternal AZT prophylaxis started in pregnancy. We hypothesize that the direction of this association may be explained by confounding by severity of disease and may not be a true effect of ART exposure. PMTCT policy during the study period recommended ART initiation only for women with CD4 cell counts ≤ 350 cells/mm^3^ and AZT for those with higher CD4 cell counts [24]. As expected women who initiated ART pre-conception had the lowest median CD4 cell count and highest rates of TB and syphilis in our study population. The small mean WAZ  profile difference between the pre-conception ARV and AZT exposure groups was attenuated in the models adjusting for confounders. Our data showed no association between mean WAZ and timing of ART initiation. The mean LAZ profile also did not vary between exposure groups. These findings are consistent with data from other South African studies showing that birthweight [34, 35] and postnatal growth [35, 36] outcomes did not vary with fetal exposure to maternal ART, but contrast with data from Ethiopia [37]. Taken together these data provide supporting evidence for initiating pregnant WLHIV immediately on ART, in line with current PMTCT policy guidelines [38].

Our data highlight other important non-HIV specific factors that are associated with growth of CHEU [39]. Mean WAZ was positively associated with some maternal (older age, higher education, and employment) and household (electricity and food security) factors. These findings support global evidence that, in addition, to nutrition-specific solutions [40], indirect non-health-care sector interventions, such as household food security, are crucial for improving child health outcomes [41]. Mean WAZ was also positively associated with male sex. Given that Z-scores are standardized for biological sex, sex may be a proxy for socio-behavioral factors, such as childcare practices, in our study sample. Our data show that maternal TB co-infection was associated with lower mean WAZ. Pregnant WLHIV have a higher likelihood of being co-infected with TB, which is associated with adverse birth outcomes such as LBW [42]. Prevention and management of other infectious diseases is therefore crucial, particularly in pregnant WLHIV. Optimal breastfeeding in the first few months of life is also important for child growth and development [43]. Our finding that breastfeeding is associated with lower mean WAZ has been previously observed among South African children [44]. This observation is likely due to earlier solid food introduction by women who do not breastfeed, which is associated with higher weight velocity and related overnutrition in children [44]. Mean WAZ also differed by geographical location with provinces such as Free State, Gauteng, Northern Cape, North West and Western Cape showing a lower mean WAZ than Eastern Cape. These unexpected observations may partly be explained by geospatial variation in the sampling participants within the provinces. In some provinces participants were sample from rural facilities while in others they were recruited from urban clinics. Findings may also reflect differences in access to healthcare and social protection services, poverty levels, hunger etc. within the provinces which would affect provincial estimates. Our observations corroborate data from nationally-representative studies [45–47]. Similar associations were observed for mean LAZ with some slight differences.

Our study has several strengths. First, this is the first study reporting on 18-month postnatal growth patterns of CHEU from a national sample in South Africa. This enabled us to assess the relationship between growth and individual-level factors and to describe growth across geographical locations. Second, study data were collected before universal ART roll-out, enabling us to compare growth of children by fetal exposure exposed to ART, AZT prophylaxis and unmanaged HIV. This comparison is important as the PMTCT programme still has HIV testing and treatment gaps that drive pediatric HIV infections and other adverse outcomes [2, 48]. Third, we used robust lab-confirmed HIV antibody and virological tests to ascertain infant HIV exposure and infection status respectively [49, 50]. Fourth, availability of rich socio-demographic, maternal and infant data enabled us to explore effects of these non-HIV related factors on CHEU growth in addition to in-utero ARV exposure. Fifth, these data, from a national cohort in a middle-income setting, add to the sparse data from large studies in these settings.

Our study has some limitations. First, as adverse perinatal outcome risk may vary by ART drug combinations [51], lack of much variability in the ART regimens used over this period precluded our ability to assess relationships between exposure to specific ARV drugs and child growth. Hence, while our study provides data regarding the effects of Nevirapine-based ART, they may not be generelisable to current Efavirenz- or Dolutegravir-based ART regimens. Second, as maternal ARV use was self-reported, there is a small chance of non-differential misclassification, albeit unlikely as women on AZT in our study had higher median CD4 cell counts than women on ART. Third, we could not adequately adjust for CD4 cell count because these data were missing for 37% participants, reflecting gaps in routine service delivery. We also had missing data for participants who missed all postnatal study visits. Fourth, viral load data were not collected. We also did not collect information on the assessment method used to ascertain gestational age. Given that most public health facilities in South Africa use the last menstrual period method, with ultrasound reserved for complicated pregnancies, we speculate that our data was subject to some measurement bias. Fifth, although feeding data were collected at all timepoints, we used 6-weeks feeding in models because of high rates of mixed feeding from 3 months. Sixth, there is likely bias by indication in our growth comparison by our ARV exposure variable, as ART initiation was CD4 cell count based. Seventh, the primary study was designed to generate national estimates, limiting inter- and intra-provincial growth comparisons. Eighth, the study had no control group of children who are HIV-unexposed, precluding comparisons against background growth patterns. Ninth, there is possible selection bias as the primary study excluded sick infants and those that died before 6 weeks of age. These infants could have been more likely to have adverse birth (e.g. preterm birth) and postnatal (e.g. stunting) outcomes. Tenth, as the definition for newly-infected mothers was partly based on self-reported HIV maternal status during pregnancy, there is possibility some participants in this exposure group may have been misclassified. We believe the probability of misclassification is small as most of these women still thought they were still HIV negative at the 4–8-weeks postpartum. Therefore, most of them had not gone for HIV-related healthcare by that timepoint.

## Conclusions

In conclusion, our national cohort study showed postnatal growth of CHEU did not vary by fetal exposure to maternal ARVs. Together with data from other studies [34–36], these findings provide supporting evidence for current recommendations about the importance of ensuring that all pregnant WLHIV are on ART. Given that the goal is to ensure that children thrive and reach their potential, longer-term monitoring for potential neurodevelopmental or other health outcomes among CHEU is required. Such monitoring should include non-HIV related maternal, child and socio-demographic risk factors for poorer child growth, which were associated with growth faltering in our data. These factors can only be mitigated through multi-sectoral collaboration and by implementing nutrition-sensitive and -specific interventions tailored for local contexts.

## Supplementary Information

**Additional file 1: Box 1**. Anthropometry data cleaning criteria. **Figure S1.** Directed Acyclic Graph representing the hypothesized relationships, 2012–2014, South Africa. **Figure S2.** Study cohort profile of HIV exposed uninfected infants from 6-weeks to 18-months postpartum, 2012–2014, South Africa. **Table S1.** Proportion of underweight children from 6-weeks to 18-months postpartum by in-utero antiretroviral exposure status, 2012–2014, South Africa. **Table S2.** Proportion of stunted children from 6-weeks to 18-months postpartum by in-utero antiretroviral exposure status, 2012–2014, South Africa. **Table S3.** Frequency of maternal antiretroviral treatment over time by baseline maternal antiretroviral categories, 2012–2014, South Africa

## Data Availability

The datasets generated and/or analyzed during the current study are not publicly available but are available from the corresponding author on reasonable request.

## Abbreviations

Ab: Antibody
ANC: Antenatal care
ARV: Antiretroviral
ART: Antiretroviral treatment
AZT: Azidothymidine
EIA: Enzyme immunoassay
GEE: Generalized estimation equation
HEU: HIV-exposed uninfected
HIV: Human immunodeficiency virus
LAZ: Length-for-age Z-score
LBW: Low birth weight
PCR: Polymerase chain reaction
PMTCT: Prevention of Mother to Child Transmission of HIV
PTB: Preterm birth
SAPMCTE: South African Prevention of Mother to Child Transmission of HIV Evaluation
SD: Standard deviation
SGA: Small for gestational age
TB: Tuberculosis
WAZ: Weight-for-age Z-score
WHO: World health organization
WLHIV: Women living with HIV
