# Supplementary material for: Impact of antenatal antiretroviral drug exposure on the growth of children who are HIV-exposed uninfected: the national South African Prevention of Mother to Child Evaluation cohort study
Source: BMC Infect Dis. 2022 Dec 6;22:908. doi: 10.1186/s12879-022-07847-9 (PMC9724324; doi:10.1186/s12879-022-07847-9)
Supplement: Supplementary file 1 — Additional file 1: Box 1. Anthropometry data cleaning criteria. Figure S1. Directed Acyclic Graph representing the hypothesized relationships, 2012–2014, South Africa. Figure S2. Study cohort profile of HIV exposed uninfected infants from 6-weeks to 18-months postpartum, 2012–2014, South Africa. Table S1. Proportion of underweight children from 6-weeks to 18-months postpartum by in-utero antiretroviral exposure status, 2012–2014, South Africa. Table S2. Proportion of stunted children from 6-weeks to 18-months postpartum by in-utero antiretroviral exposure status, 2012–2014, South Africa. Table S3. Frequency of maternal antiretroviral treatment over time by baseline maternal antiretroviral categories, 2012–2014, South Africa [file 12879_2022_7847_MOESM1_ESM.docx]

**SUPPLEMENTARY MATERIAL**

**Box 1: Anthropometry data cleaning criteria**

| Anthropometric measurements and Z-scores were checked if the following criteria were met: 1) birthweight-for-gestational Z-score <-6 or >6 ; 2) WAZ less than -6 or >5, LAZ <-6 or >6, WLZ <-5 or >5, WLZ>3 or LAZ<-3; 3) >2cm decrease in length between consecutive visits; 4) extreme differences in LAZ between consecutive visits, for example 6-week LAZ<2 and 3-month LAZ >2.5 ; 5) LAZ or WLZ differences > 2.5 or <-2.5 Z-score between 6-week and 3-month visit or >4 or <-4 Z-score between consecutive visits after the 3-month visit. Values were set to missing if no plausible explanation was established. |
| --- |

Child age

CD4 count

in-utero ART exposure

LBW

PTD

SGA

Maternal age

HH water, electricity, flush toilet, brick house

Maternal education

ANC attendance

Complementary feeding

Breast feeding

Postnatal growth

Syphilis infection

HH food insecurity

Parity

TB infection

Delivery by C/S

Child morbidity

Child hospitalization

CD4(confounder by indication): CD4 is both an indication for ARV treatment and risk factor for birth outcomes. Therefore, adjusting for CD4 count may introduce confounding by indication. Adjusting (e.g. stratifying) any variables that are affected by the exposure of interest (e.g. intermediates such as birthweight) and share common causes with the outcome (e.g. the unmeasured variables) would result in a spurious association between prenatal exposure (ART) and postnatal outcome (growth) due to selection bias

Figure 1: Directed Acyclic Graph **representing the** hypothesized relationships, **2012-2014, South Africa**

**Unmeasured variables: smoking, alcohol use, viral load, maternal BMI, GWG**

**4 PCR+**

**Figure 2: Study cohort profile of** c**hildren who are HIV-exposed uninfected from 6-weeks to 18-months postpartum, 2012-2014, South Africa**

Definitions: ARV: Antiretroviral; LAZ: length-for-age Z-score; m: month; PCR: polymerase chain reaction; WAZ: weight-for-age Z-score; w: week

**Table 1: Proportion of underweight children from 6-weeks to 18-months postpartum by in-utero antiretroviral exposure status, 2012-2014, South Africa**

| **Underweight n/N (%)** | **Pre-conception ART N=617** | **Post-conception ART N=782** | **AZT N=879** | **None N=189** | **Newly infected mothers N=59** | **Total N=2526** | **p-value*** |
| --- | --- | --- | --- | --- | --- | --- | --- |
|  | **n/N (%)** | **n/N (%)** | **n/N (%)** | **n/N (%)** | **n/N (%)** | **n/N (%)** |  |
| 6 weeks | 81/583 (13.89) | 89/735 (12.11) | 102/836 (12.20) | 28/165 (16.97) | 8/51 (15.69) | 308/2370 (13.00) | 0.40 |
| 3 months | 41/434 (9.45) | 46/532 (8.65) | 45/575 (7.83) | 16/108 (14.81) | 1/5 (20.00) | 149/1654 (9.01) | 0.18 |
| 6 months | 37/449 (8.24) | 36/505 (7.13) | 27/548 (4.93) | 7/101 (6.93) | 1/16 (6.25) | 108/1619 (6.67) | 0.33 |
| 9 months | 30/434 (6.91) | 32/504 (6.35) | 24/523 (4.59) | 8/100 (8.00) | 0/27 (0) | 94/1588 (5.92) | 0.28 |
| 12 months | 35/438 (7.99) | 24/495 (4.85) | 27/508 (5.31) | 8/95 (8.42) | 1/33 (3.03) | 95/1569 (6.05) | 0.19 |
| 15 months | 38/439 (8.66) | 33/488 (6.76) | 29/530 (5.47) | 5/106 (4.72) | 2/34 (5.88) | 107/1597 (6.70) | 0.32 |
| 18 months | 30/464 (6.47) | 27/534 (5.06) | 23/568 (4.05) | 5/106 (4.72) | 1/38 (2.63) | 86/1710 (5.03) | 0.46 |

*Pearson's chi-squared test

Definitions: ART: antiretroviral therapy (Maternal ART regimens generally consisted of Tenofovir, Lamivudine or Emtricitabine and Nevirapine); AZT: Azidothymidine; None, children with no fetal ARV exposure

**Table 2: Proportion of stunted children from 6-weeks to 18-months postpartum by in-utero antiretroviral exposure status, 2012-2014, South Africa**

| **Stunted n/N (%)** | **Pre-conception ART N=617** | **Post-conception ART N=782** | **AZT N=879** | **None N=189** | **Newly infected mothers N=59** | **Total N=2526** | **P-value*** |
| --- | --- | --- | --- | --- | --- | --- | --- |
|  | **n/N (%)** | **n/N (%)** | **n/N (%)** | **n/N (%)** | **n/N (%)** | **n/N (%)** |  |
| 6 weeks | 84/223 (37.67) | 93/326 (28.53) | 108/349 (30.95) | 30/67 (44.78) | 11/31 (35.48) | 326/996 (32.73) | 0.04 |
| 3 months | 101/346 (29.19) | 118/439 (26.88) | 145/469 (30.92) | 24/84 28.57) | 2/3 (66.67) | 390/1341 (29.08) | 0.42 |
| 6 months | 125/431 (29.00) | 145/483 (30.02) | 153/533 (28.71) | 37/100 (37.00) | 2/15 (13.33) | 462/1562 (29.58) | 0.30 |
| 9 months | 136/426 (31.92) | 140/492 (28.46) | 143/506 (28.26) | 29/96 (30.21) | 5/24 (20.83) | 453/1544 (29.34) | 0.61 |
| 12 months | 137/427 (32.08) | 154/480 (32.08) | 145/503 (28.83) | 27/92 (29.35) | 10/31 (31.25) | 473/1534 (30.83) | 0.79 |
| 15 months | 158/431 (36.66) | 159/479 (33.19) | 163/523 (31.17) | 37/107 (34.58) | 11/35 (31.43) | 528/1575 (33.52) | 0.50 |
| 18 months | 158/461 (34.27) | 171/529 (32.33) | 175/564 (31.03) | 38/107 (35.51) | 12/38 (31.58) | 554/1699 (32.61) | 0.80 |

*Pearson's chi-squared test

Definitions: ART: antiretroviral therapy (Maternal ART regimens generally consisted of Tenofovir, Lamivudine or Emtricitabine and Nevirapine); AZT: Azidothymidine;; None, children with no fetal ARV exposure

**Table 3: Frequency of maternal antiretroviral treatment over time by baseline maternal antiretroviral categories, 2012-2014, South Africa**

| **Covariate** | **Total N=2526** | **Pre-conception ART N=617** | **Post-conception ART N=782** | **AZT N=879** | **None N=189** | **Newly infected mothers N=59** |
| --- | --- | --- | --- | --- | --- | --- |
|  | **n/N (%)** | **n/N (%)** | **n/N (%)** | **n/N (%)** | **n/N (%)** | **n/N (%)** |
| **Mother on ART** |  |  |  |  |  |  |
| 6 weeks | 1415/2467 (57.36) | 617/617 (100) | 782/782 (100) | 16/874 (1.83) | 0/189 (0) | 0/5 (0) |
| 3 months | 877/1622 (54.07) | 388/425 (91.29) | 415/505 (82.18) | 59/577 (10.23) | 13/109 (11.93) | 2/6 (33.33) |
| 6 months | 871/1510 (57.68) | 381/416 (91.59) | 379/463 (81.86) | 84/515 (16.31) | 22/100 (22.00) | 5/16 (31.25) |
| 9 months | 869/1457 (59.64) | 374/406 (92.12) | 375/463 (82.17) | 96/472 (20.34) | 19/92 (20.65) | 5/24 (20.83) |
| 12 months | 848/1426 (59.47) | 351/389 (90.23) | 364/443 (82.17) | 99/476 (20.80) | 26/91 (28.57) | 8/27 (29.63) |
| 15 months | 846/1394 (60.60) | 348/384 (90.63) | 339/412 (82.28) | 120/466 (25.75) | 29/101 (28.71) | 10/31 (32.26) |
| 18 months | 878/1440 (60.97) | 359/392 (91.58) | 345/438 (78.77) | 128/484 (26.45) | 35/94 (37.23) | 11/32 (34.38) |

Definitions: ART: antiretroviral therapy (Maternal ART regimens generally consisted of Tenofovir, Lamivudine or Emtricitabine and Nevirapine); AZT: Azidothymidine; None, children with no fetal ARV exposure
